# Single Buried Intramedullary K-Wire Fixation in Nonthumb Metacarpal Shaft Fractures with Immediate Postoperative Mobilization without Any Immobilization

**DOI:** 10.1155/2023/1439011

**Published:** 2023-10-16

**Authors:** Wuttipong Siriwittayakorn, Nath Adulkasem, Pichet Sangthongsil, Wasapol Pitiguagool, Wattanai Atthakorn, Kraisong Watatham, Wichit Siritattamrong

**Affiliations:** ^1^Department of Orthopaedics, Nakornping Hospital, Chiang Mai, Thailand; ^2^Department of Orthopaedics, Faculty of Medicine, Siriraj Hospital, Bangkok, Thailand; ^3^Chularat 3 Hospital, Bang Phli, Samut Prakarn, Thailand; ^4^Department of Orthopaedics, Faculty of Medicine, Prince of Songkla University, Songkhla, Thailand

## Abstract

**Objective:**

This study aims to evaluate the outcomes of single intramedullary K-wire fixation in nonthumb, metacarpal shaft fractures with immediate postoperative hand mobilization without any immobilization.

**Method:**

This is a retrospective case series conducted from January 2019 to December 2022. We included patients with closed, simple transverse, or short oblique metacarpal shaft fracture treated with single, 1.4 mm, intramedullary K-wire fixation. Gentle postoperative range of motion exercise was encouraged in every patient without any hand, finger, or wrist motion restriction material. Clinical outcomes were evaluated with total active flexion; grip strength; disability of arm, shoulder, and hand (DASH) score; and the American Society for Surgery of the Hand Total Active Flexion (ASSH TAF) score.

**Results:**

This study included 34 patients, 25 males and 9 females with a mean age of 33.14 years (ranging 18–59). A total of 43 metacarpal shafts were treated. The mean DASH score at two and 6 weeks postoperative was 41.5 (ranging 19.16–60.34) and 9.58 (ranging 0.83–23.27). The mean final DASH score at last follow-up was 3.48 (ranging 0–8.33). Mean TAF at 2 weeks postoperative, 6 weeks postoperative, and at final follow-up was 203.8 (ranging 185–240), 238.2 (ranging 220–270), and 259.25 (ranging 240–270) degrees, respectively. The mean grip strength of the injured hand was 66.14 and 86.1% of the uninjured hand at 6 weeks and 3 months postoperative. There was no nonunion, malrotation, or infection. In conclusion, single intramedullary K-wire fixation gives excellent outcomes in the treatment of single or multiple, simple, metacarpal shaft fractures without the need of postoperative immobilization.

## 1. Introduction

Fracture of the metacarpal and phalanges are the most common fractures of the upper extremity [1, 2]. The estimated incidence of metacarpal fracture is 13.6 per 100,000 person/year [3]. Although hand fractures can be successfully treated by nonoperative treatment, several complications (e.g., malunion and stiffness due to prolonged immobilization) are common in this treatment method [1, 4]. Open reduction and internal fixation with plate and screw could provide excellent fracture reduction and stability; this operative treatment method has a higher risk of soft-tissue damage, adhesion, or infection [1, 5–8]. Hence, minimal invasive operative treatments have gained popularity as they promote bone healing with minimal soft-tissue dissection and limited fracture immobilization duration [1, 4, 6, 7, 9, 10].

Antegrade intramedullary (IM) Kirschner wire (K-wire) fixation is a well-known, minimally invasive, treatment option in metacarpal fracture [1, 5, 10]. It has been proven that IM K-wire fixation is not inferior to plate and screw fixation in treating nonthumb metacarpal fractures [5–7, 10–13]. The concept of multiple IM K-wire fixations has been proposed [14–17]; however, at present, more studies have reported the sufficiency of a single IM K-wire fixation [18–22].

There is still no consensus on the postoperative management of metacarpal fracture. The rehabilitation protocol varies and depends on the preference and experience of each institute. Several authors have lessened the immobilization material and promoted early mobilization after K-wire fixation [14, 15, 18–20, 23–28]. Moreover, recent evidence revealed that early mobilization of fingers after the injury causes significant consequences in only a few patients who underwent nonoperative treatment [29–32]. These studies raise the question of the necessity of postoperative hand immobility.

Therefore, our objective was to evaluate outcomes along with the complications of immediate postoperative hand mobilization in metacarpal shaft fracture patients who underwent single buried antegrade IM K-wire fixation without postoperative immobilization. We hypothesized that immediate postoperative hand mobilization can be initiated after single IM K-wire fixation in metacarpal shaft fracture and the fracture unite without serious complications.

## 2. Patients and Methods

### 2.1. Study Population

We conducted a retrospective case series of patients diagnosed with metacarpal shaft fracture to evaluate the outcome of single, buried, antegrade IM K-wire fixation within 2 weeks of the initial injury. The study was conducted between January 2019 and December 2022. The inclusion criteria were as follows: (1) closed simple transverse or short oblique metacarpal shaft fractures and (2) unacceptable deformities [13, 18] that could not be treated by nonoperative treatment or patients who could not withstand conservative treatment with a slab or cast immobilization. We included patients with either single or multiple metacarpal shaft fractures. We excluded patients with comminuted, spiral, and long oblique metacarpal shaft fracture which were not appropriate for IM K-wire fixation [1, 5]. Patients with concomitant injury that would preclude early hand mobilization (i.e., severe head injury) were also excluded from this study. The Institutional Review Board approved our study protocol (num 027/65).

### 2.2. Operative Technique

Surgery was performed under wrist block which allows for intraoperative active hand motion to ensure proper fracture reduction and fixation. The patient was placed in the supine position with the pronated and extended arm on a hand table. The tourniquet was inflated to control the bleeding. The procedure was performed under fluoroscopic guidance.

A 1 cm skin incision was made, starting from the base of the affected metacarpal and continued proximally. Subcutaneous vessels and tendons were retracted and protected using a Senn retractor while exposing the metacarpal base. The dorsal cutaneous branch of ulnar nerve (DCBUN) should be identified and protected when operating on the fourth or fifth metacarpal. The 2.0 mm K-wire was inserted into a universal T-handle chuck. This K-wire was used as an awl to create an entry hole at the cancellous bone of the metacarpal base, just distal to the carpometacarpal joint. This entry hole should always be in line with the central axis of the operated metacarpal.

**Figure 1:**
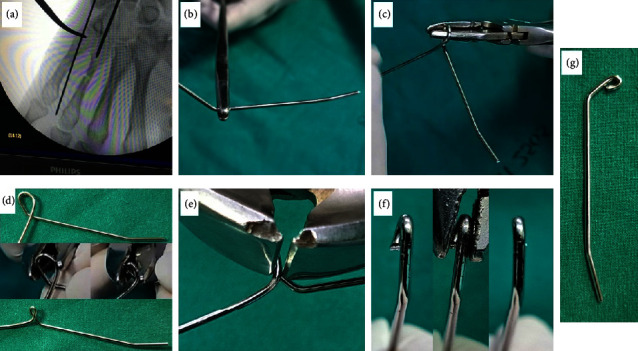
The steps in the K-wire prebending: (a) how to measure the length of the implant, (b) using the blunt end of the K-wire as an implant tip, the tip was bent up about five degrees and the proximal part of the K-wire was bent up about 45 degrees, (c) the loop was made by twisting the wire around the plier, (d) the plier is used to further compress the loop diameter, (e) cut and remove the remaining K-wire, (f) the cut proximal K-wire end was twisted to align with the implant, thus preventing the present of any sharp edge that could irritate the soft tissue, and (g) the final shape of the implant.

Typically, a 1.4 mm K-wire is chosen. Before bending, the pointy tip of the K-wire is cut to prevent any potential injury to the surgeon. The blunt end of the K-wire will be introduced into the medullary canal to avoid metacarpal head perforation. Once the metacarpal fracture is reduced, the K-wire is placed in position with the assistance of an image intensifier. The distal tip of the K-wire is positioned at the subchondral bone of the distal metacarpal metaphysis. The length of the implant is determined by measuring the length from the tip of the K-wire to the entry point. At the entry point, the K-wire is bent up about 45 degrees and its proximal end is twisted into a loop. Each patient's soft tissue thickness will determine the position of the loop. This loop should be located in the subcutaneous tissue when the implant was properly introduced into the metacarpal (Figure 1).

The implant was introduced into the medullary canal through the previously made entry point. Any malrotation must be corrected before passing the K-wire through the fracture site. The K-wire was pushed to engage firmly into the subchondral bone. Intraoperative active hand motion under the image intensifier was always performed to evaluate any displacement from early hand motion. The implant was buried under the skin and the skin was closed using a nonabsorbable suture.

### 2.3. Postoperative Protocol

Immediate mobilization of the wrist and all fingers was initiated after surgery without any motion restriction instrument. During the first two weeks, with no specific rehabilitation protocol from the surgeon or hand therapist, only gentle flexion and extension of all metacarpophalangeal and interphalangeal joints were allowed. The patients were permitted to resume performing light daily activities, that is, the patients can use their hand in their daily activities only as pain allows. The patients were encouraged to do gentle progressive hand and finger motions to eventually achieve full range of motion (ROM). Forceful hand clenching or pinching was strictly prohibited.

The suture material was removed 2 weeks after the operation. Carefree contact with water in daily activities was allowed after the removal of the suture material. All patients were advised that no pain or discomfort should be felt in the injured site during the activities. If any discomfort is felt, the patient should discontinue that activity and rest the operated hand.

Finger malrotation was assessed clinically by observing finger scissoring at full finger flexion and extension [30]. We observed the ROM and malrotation, evaluated the grip strength and DASH score, and obtained a repeat plain radiograph of the hand at 2 and 6–8 weeks then 3 months after the operation. We defined radiographic bone healing as a present of bony coalition at the fracture site [33]. If clinical signs of bone union and radiographic bone healing were observed, the K-wire was removed. The average time for implant removal was approximately 6 weeks. We removed the K-wire under local anesthesia in an out-patient department. Grip strength and total active flexion (TAF) of the operated hand were compared to the uninjured hand. When multiple metacarpals were broken, data of the finger with worse TAF were recorded.

Functional outcomes were assessed at 2 weeks, 6 weeks, and 3 months postoperative by using the American Society for Surgery of the Hand (ASSH) TAF score [34], disability of arm shoulder and hand (DASH) score [35] and TAF compared to the uninjured hand (%TAF). ASSH TAF was determined to be excellent (>220^o^ TAF), good (120–80^o^ TAF), or poor (<80^o^ TAF).

### 2.4. Statistical Analysis

All statistical analyses were performed using STATA 16 (StataCorp LLC, College Station, TX, USA). Data distribution was evaluated with the Shapiro–Wilk test. Continuous data with normal distribution were demonstrated using mean ± standard deviation (SD) and median with interquartile range (IQR) for non-normal distribution. Categorical data were presented with count and percentage. Inferential statistics were determined using paired *t*-test, Wilcoxon signed rank test, and Fisher's exact test according to data characteristics. The level of statistical significance was set at *p*  <  0.05.

## 3. Results

This study included 34 patients, 25 males and 9 females with a mean age of 33.52 years (ranging 18–59). There were 9 patients with multiple metacarpal fracture. A total of 43 metacarpal shafts were treated. Demographic data of the patients are shown in Table 1.

The mean overall clinical follow-up period was 97.08 days (ranging 40–154). Healing was obtained in all patients. A sign of radiographic bone union was observed at a mean of 47.3 days (range 37–56). No infection was observed.

**Figure 2:**
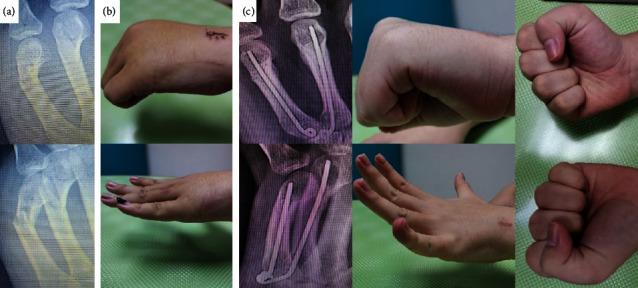
A 19-year-old male, with fracture of the fourth and fifth metacarpal shafts: (a) preoperative radiographs, (b) 2 weeks postoperative range of motion, (c) at 6 weeks after the operation, the patient's radiographs showed a sign of bone union. He regained excellent ASSH TAF score. His TAF of his left ring finger was 265 degrees (normal: 270). His grip strength was 71.15% of the contralateral hand. His DASH score was 14.16. He had lost to follow-up after implant removal.

Among 34 patients, three patients satisfied with the treatment result and declined further appointment after K-wire extraction. Four patients were lost after implant removal. All 7 patients with short follow-up showed sign of bone union and achieved excellent ASSH TAF score (Figure 2).

There are 27 patients, and 32 metacarpals that were eligible for longer follow-up. The mean DASH score at 2 and 6 weeks postoperative was 41.5 (ranging 19.16–66.6) and 9.58 (ranging 0.83–23.27). The mean final DASH score at last follow-up was 2.66 (ranging 0–8.33). Mean TAF at 2 weeks postoperative, 6 weeks postoperative, and at final follow-up was 203.8 (ranging 185–240), 238.2 (ranging 220–270), and 259.25 (ranging 240–270) percent, respectively. Mean %TAF at 2 weeks postoperative, 6 weeks postoperative, and at final follow-up was 77.02 (ranging 68.51–88.88), 89.99 (ranging 83.01–100), and 97.97 (ranging 90.56–100) percent, respectively. Hand grip strength was assessed by digital hand grip strength dynamometer compared to the uninjured hand. The mean grip strength of the injured hand compared to the uninjured hand at 6 weeks postoperative and the last appointment were 66.96 (51.83–90.27) and 86.1 (72.79–97.87) percent, respectively (Table 2).

Scissoring of finger during full fingers' flexion had never been observed throughout the treatment course. There was no infection, stiffness, tendon irritation, or metacarpal head perforation observed. Five patients with fifth metacarpal fracture reported minimal postoperative paresthesia on the dorso-ulnar side of the injured hand. All patients' symptoms resolved within 3 months after surgery. One patient with fifth metacarpal shaft fracture had a dorsal callus bump at the fracture site.

## 4. Discussion

IM K-wire fixation in metacarpal fracture is a well-known treatment. There are many described techniques and postoperative protocols for this procedure. Abulsoud et al. were the first to report a study that describes and investigates the outcomes of fixation of metacarpal shaft fractures using a single, 1.8–2.0 mm, buried K-wire [18]. In our technique, due to a smaller body configuration of the Asian population, it is difficult to insert a 1.8 mm K-wire into the metacarpal medullary canal. Thus, we have chosen to use the 1.4 mm K-wire instead. A bigger or smaller diameter of the K-wire can be chosen based on the diameter of the medullary canal [28]. Nonetheless, by performing intraoperative active hand motion under an image intensifier and based on the findings of our study, we found that single 1.4 mm K-wire can also maintain fracture stability throughout the treatment course, even in a fifth metacarpal where the medullary canal diameter is wider than the others. However, it is of utmost importance to note that the tip of the implant must firmly engage with the subchondral bone to achieve stability and only gentle postoperative hand movement that cause no hand discomfort can be allowed until bone union.

**Figure 3:**
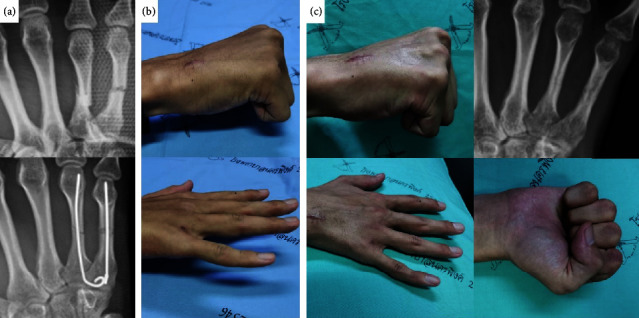
A 23-year-old male with fracture of the fourth and fifth metacarpal shafts: (a) pre and postoperative radiographs of his right hand, (b) clinical pictures of the patient's hand ROM at 6 weeks postoperative, (c) clinical pictures of his ROM at 3 months after surgery. The radiograph showed bone union. He regained full ROM. His DASH score was 5 and his grip strength was 77.46% compared to his left hand.

According to Abulsoud et al., their study population was limited only to patients with single metacarpal shaft fractures [18]. Various studies also reported outcomes of single IM K-wire fixation in metacarpal neck fractures, further supporting the concept of single IM K-wire fixation [18–22]. Nonetheless, every study on a single IM K-wire applied postoperative immobilization. Based on our findings, immediate postoperative gentle hand motion can be initiated, without the need of postoperative immobilization in patients with simple metacarpal shaft fracture. Our results support the idea of single IM K-wire fixation in metacarpal shaft fracture. Furthermore, we observed that single IM K-wire fixation is also adequate in stabilizing multiple metacarpal shaft fractures (Figure 3).

**Figure 4:**
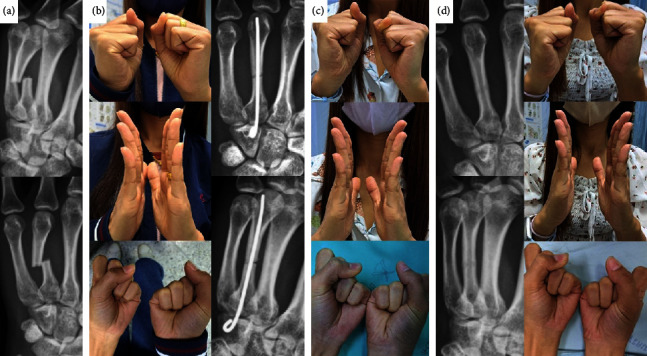
A 23-year-old female with fracture of the fourth metacarpal shaft of her left hand: (a) preoperative radiographs, (b) postoperative radiographs and clinical pictures of her hand motion at 2 weeks, (c) the patient regained full ROM at 6 weeks after the operation, and (d) uneventful bone healing and excellent functional outcomes were observed at 5 months. Her grip strength was 86.48% and the DASH score was zero. She denied further appointment.

From our findings and previous reported literature, immediate gentle postoperative ROM of the metacarpophalangeal joint (MPJ) and interphalangeal joint (IPJ) does not affect fracture healing or patient's hand function [28]. Immediate mobilization provides several advantages, including a rapid return to daily activities, fast recovery of hand ROM, and fewer complications and discomfort from prolonged immobilization [18, 23, 24, 27, 28, 30]. Our operative technique and protocol of immediate mobilization are slightly different from what was described earlier by Rocchi et al. [28]. We apply a single K-wire in every metacarpal fracture. The operation is always carried out under local anesthesia to evaluate intraoperative active hand motion. Consequently, it can be ensured that every fracture achieves stability during active hand mobilization (Figure 4). In addition, the implant is buried under the skin. By doing so, every patient reported that they can comfortably return to daily activity such as bathing and self-care after the operation.

We prefer to curl the proximal end of the K-wire into a loop and bury them under the skin to prevent tendon and soft-tissue irritation or a possibility of pin tract infection. In previously reported techniques of buried IM K-wire, the proximal end of the K-wire was frankly cut. With such a straight cut, there have been reports of tendon rupture, skin irritation, pain, and stiffness [12, 22, 36]. Even the surgeon cut the K-wire at the periosteum level to reduce the overlying soft-tissue irritation, late K-wire migration, or pain and stiffness from soft-tissue irritation can occur [14, 26, 36]. Moreover, the K-wire that was cut too close to the bone is difficult to remove [25, 36, 37]. Our patients never report pain or stiffness from the buried implant. Observing from our treatment results, we believe that our technique could reduce soft-tissue complications such as skin or tendon irritation. In addition, the twisted proximal end of the K-wire can be easily identified and removed.

In our protocol, an adequate intensity of postoperative mobilization must be tailored according to the patient's tolerability. The patient is advised to limit the affected hand activity if there is any pain at the fracture site. As a result, the patient's rehabilitation intensity increased inversely to pain. Subsequently, the patients will gradually regain their normal ROM along with the fracture healing.

There were no cases of malrotation after gentle finger flexion and extension in simple metacarpal shaft fracture with single IM K-wire fixation. According to previous studies, fracture rotational malalignment was greatly influenced by the integrity of the deep transverse metacarpal ligament (DTML) [18, 19, 30, 31, 38–42]. DTML is a narrow fibrous band that extends in a radio-ulnar direction palmar to the MCP joint of the second to fifth metacarpal bones [38]. These ligaments have a complex interconnection, via stiff fibers, to the palmar aponeurosis, connective tissue septa, and collagen fiber nets of the skin [38, 39]. DTML maintains the metacarpal arch, thus reducing deformation during clenching or holding movements [38, 43].

Severe rotation deformity can be seen only if the DTML is transected, attenuated, or ruptured [30, 40, 41, 43]. DTML plays a significant role in preventing rotational deformity [30, 43]. No malrotation was seen in the metacarpal fracture with no initial rotatory displacement even after early mobilization of the DTML was intact [31]. Rotatory displacement after the flexible fixation of metacarpal fracture is rare [7, 18, 19, 21, 23, 24, 26, 40, 44, 45]. Though we are unable to measure an exact malrotation of the metacarpal in our population, finger scissoring had never been noticed. Early postoperative mobilization, from our experience and many reported literature studies, does not produce rotational deformity [14, 15, 19, 21–26, 31].

**Figure 5:**
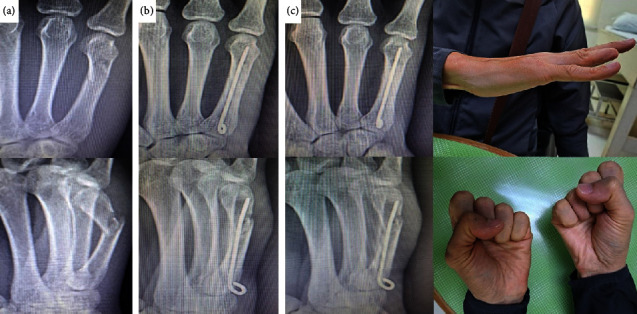
A 51-year-old male with fracture of the fifth metacarpal shaft of the right hand: (a) preoperative radiographs, (b) postoperative radiographs at 2 weeks after surgery, and (c) at 6 weeks postoperative, the patient had dorsal callus bump over the fracture site. He had 15 degrees extension lag, but with full finger flexion. His grip strength was 82.85% and his DASH score was 3.33. He was satisfied with the treatment result and denied further appointment.

Complications of single IM K-wire fixation in metacarpal shaft fractures include stiffness, nonunion, and metacarpal head perforation [18]. Though we did not observe these complications in our population, we did notice other complications. In patients with fifth metacarpal shaft fractures, DCBUN irritation can be observed. In our patient, hyposthesia also recovered within 3 months after surgery as stated by She et al. [22]. There can be a dorsal callus formation at the fracture site which may limit full finger extension. Nonetheless, this complication was observed in only one of our patients, who had full finger flexion and excellent return of function at 6 weeks postoperative. The patient declined further appointment, thus we were unable to observe a long-term result of this complication (Figure 5). From the previously reported literature, we supposed that extension lag would resolve within one year after surgery [31].

To the best of our knowledge, we are the first to report outcomes of metacarpal shaft fracture fixation using single buried K-wire, with immediate postoperative hand motion without any immobilization. However, there are some drawbacks to this study and the surgical protocol. Firstly, the follow-up time was relatively short. We attempted to follow our patient for up to one year, but every patient declined further appointments when the function of their operated hand was comparable to their contralateral hand. Secondly, there is also a flaw in exposing surgeons, personnel, and the patient to radiation during the operation. After bone union, the patient requires a second operation to remove the implant. Lastly, this is a retrospective case series without comparison with other techniques. Therefore, a larger prospective comparative study with a longer follow-up period is needed to further investigate these outcomes.

## Figures and Tables

**Table 1:** Demographic data.

Characteristics	Value
*Age (years)*	
Minimum	18
Maximum	59
Mean (SD)	33.52 (12.56)

*Gender*	
Male	25
Female	9

*Involved bone*	
Second metacarpal	4
Third metacarpal	3
Fourth metacarpal	14
Fifth metacarpal	22

**Table 2:** Results.

Characteristics	2 weeks PO	6 weeks PO	Final
*DASH score*			
Minimum	19.16	0.83	0
Maximum	66.6	23.27	8.33
Mean ± SD	41.5 ± 12.92	9.58 ± 5.69	2.66 ± 2.64

*%TAF*			
Minimum	68.51	83.01	90.56
Maximum	88.88	100	100
Mean ± SD	77.02 ± 5.14	89.99 ± 4.76	97.97 ± 2.76

*%Grip strength*			
Minimum	—	51.83	72.79
Maximum	—	90.27	97.87
Mean ± SD	—	66.96 ± 8.96	86.1 ± 6.25

PO: postoperative; DASH: disability of arm shoulder and hand; TAF: total active flexion.

## Data Availability

The datasets used and analyzed during the current study are available from the corresponding author on request.
